# Intensive therapy after botulinum toxin in adults with spasticity after stroke versus botulinum toxin alone or therapy alone: a pilot, feasibility randomized trial

**DOI:** 10.1186/s40814-018-0276-6

**Published:** 2018-05-22

**Authors:** Natasha A. Lannin, Louise Ada, Tamina Levy, Coralie English, Julie Ratcliffe, Doungkamol Sindhusake, Maria Crotty

**Affiliations:** 1 School of Allied Health (Occupational Therapy), La Trobe University, Melbourne, Australia; 2 Occupational Therapy Department, The Alfred, 55 Commercial Road, Prahran, Victoria Australia; 3 Physiotherapy, The University of Sydney, Sydney, Australia; 4 Repatriation General Hospital, Flinders University, Adelaide, Australia; 5 School of Health Sciences and Priority Research Centre for Stroke and Brain Injury, The University of Newcastle, Newcastle, Australia; 6 Institute for Choice, UniSA Business School, University of South Australia, Adelaide, Australia; 7 Clinical Excellence Commission New South Wales, Sydney, Australia; 8 Repatriation General Hospital; Flinders University, Adelaide, Australia

**Keywords:** Muscle spasticity, Botulinum toxins, Rehabilitation, Stroke, Occupational therapy, Physical therapy

## Abstract

**Background:**

Botulinum toxin-A is provided for adults with post-stroke spasticity. Following injection, there is a variation in the rehabilitation therapy type and amount provided. The purpose of this study was to determine if it is feasible to add intensive therapy to botulinum toxin-A injections for adults with spasticity and whether it is likely to be beneficial.

**Methods:**

Randomized trial with concealed allocation, assessor blinding, and intention to treat analysis. Thirty-seven adults (*n* = 3 incomplete or lost follow-up) with spasticity in the upper or lower limb were allocated to one of three groups: experimental group received a single dose of botulinum toxin-A plus an intensive therapy for 8 weeks, control group 1 received a single dose of botulinum toxin-A only, and control group 2 received intensive therapy only for 8 weeks. Feasibility was measured by examining recruitment, intervention (adherence, acceptability, safety), and measurement. Benefit was measured as goal achievement (Goal Attainment Scale), upper limb activity (Box and Block Test), walking (6-min walk test) and spasticity (Tardieu scale), at baseline (week 0), immediately after (week 8), and at three months (week 12).

**Results:**

Overall recruitment fraction for the trial was 37% (eligibility fraction 39%, enrolment fraction 95%). The 26 participants allocated to receive intensive rehabilitation attended 97% of clinic-based sessions (mean 11 ± 2 h) and an averaged 58% (mean 52 ± 32 h) of prescribed 90 h of independent practice. There were no study-related adverse events reported. Although participants in all groups increased their goal attainment, there were no between-group differences for this or other outcomes at week 8 or 12.

**Conclusion:**

Providing intensive therapy following botulinum toxin-A is feasible for adults with neurological spasticity. The study methods are appropriate for a future trial. A future trial would require 134 participants to detect a between-group difference of 7 points on Goal Attainment Scale *t*-scores with an alpha of 0.05 and power of 80%.

**Trial registration:**

ACTRN12612000091808. Registered 18/01/2012, retrospective

## Background

Spasticity affects approximately 20% of stroke survivors [1–4] and is thought to significantly contribute to falls after stroke [5, 6] as well as decreased activity participation [3, 4]. Unsurprisingly, higher costs are thus incurred by patients with spasticity during the first year of survival [7]. Health professionals identify that addressing spasticity is a high priority during rehabilitation [8], and there is international consensus that localized spasticity (i.e., in the upper or lower limbs) is best managed with a combination of botulinum toxin and physical therapy [9, 10]. While these consensus papers appear to agree, clinical management remains diverse [11, 12] and provides an ongoing challenge for both therapists and health services alike.

In Australia, stroke rehabilitation is guided by the Stroke Foundation clinical practice guidelines [13]. These guidelines recommend that management of moderate to severe spasticity include the use of botulinum toxin type A *in addition* to physical therapy interventions [13]. Unfortunately, clinical survey data suggests that occupational therapists and physiotherapists report providing therapy post-botulinum toxin type A injections less than a quarter of the time (an estimated 16%) [12]. This low rate of therapy provision suggests ongoing uncertainty among clinicians as to how to treat patients with spasticity. Such uncertainty is likely to stem from the lack of research studies that describe the type, frequency, intensity, and duration of therapy that is effective for people who have received botulinum toxin injections. While there are previous studies which have tested the efficacy of botulinum toxin type A for spasticity management after stroke [14–16], what remains unknown is whether or not therapy should be provided to this group of patients.

To inform best practice in the treatment and rehabilitation of spasticity in people with neurological conditions, understanding whether the suggested combined effects of using both therapy and botulinum toxin type A together is more beneficial than botulinum toxin-A alone or physiotherapy interventions alone is key. Given the lack of research in this area, a large, powered randomized controlled trial is required. In preparation for this trial, it is key to understand both the feasibility and likely effects of the interventions; therefore, the research questions posed in this pilot study were:

1. In neurological patients with spasticity, is it feasible to add intensive therapy to botulinum toxin-A injections if the therapy includes both clinic-based and home-based therapy sessions?
2. Is adding intensive therapy likely to be of any benefit to goal attainment, upper limb activity, walking, and spasticity over botulinum toxin-A alone or intensive therapy alone?

## Methods

### Design

A three-group randomized feasibility trial with concealed allocation, assessor blinding, and intention-to-treat analysis was conducted at a metropolitan rehabilitation service in Adelaide, Australia. A computer-generated randomization schedule was developed by an independent person who was remote from the area where the study occurred. Allocation was concealed from the recruiter through the use of sealed consecutively numbered opaque envelopes. Participants with spasticity were randomly allocated into one of the three groups:

- Experimental group received a single dose of botulinum toxin-A plus an 8-week intensive rehabilitation program
- Control group 1 received a single dose of botulinum toxin-A only
- Control group 2 received an 8-week intensive rehabilitation program only.

**Fig. 1:**
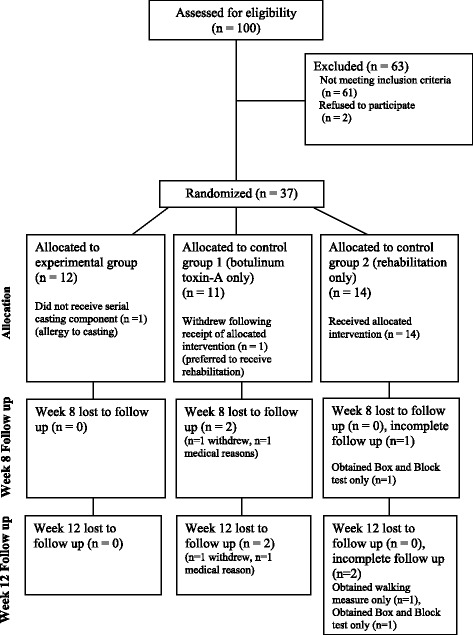
CONSORT diagram showing the flow of participants through each stage of the trial

Outcomes were measured at baseline (week 0), immediately after the intervention (week 8), and beyond the intervention (week 12). The week 12 assessment was included to reflect outcomes once the effect of the botulinum toxin-A had worn off. The design of the trial is presented in Fig. 1. Outcome measures were collected by a physiotherapist who was trained in the procedures and blinded to group allocation. To maintain blinding, participants were asked not to discuss any aspect of the trial with the assessor. University and hospital human research ethics committees approved this study, and all participants gave informed consent before data collection began.

### Participants and therapists

Participants were included in the study if they had been referred to a spasticity clinic for management of spasticity of the upper and/or lower limb as indicated by a score of 2 or more on the modified Ashworth scale [17], were at least 1 month post-neurologically impaired, were medically stable, and were able to understand simple instructions (Mini Mental State Examination score ≥ 21, [18]. Patients were excluded if they had received botulinum toxin-A in the previous 5 months, had a known allergy or hypersensitivity to botulinum toxin-A, had another significant health conditions (such as arthritis), were pregnant or breastfeeding [19], or were unable to attend the hospital for clinic appointments.

Physiotherapists and occupational therapists providing the intensive rehabilitation program all had experience in neurological rehabilitation and were trained in task-specific motor training, electrical stimulation, and serial casting prior to the study commencement.

### Intervention

The experimental group received botulinum toxin-A injections by an experienced rehabilitation physician. Muscles injected were determined by the physician based on whether they appeared to contribute to abnormal limb position and impair use of the limb [20]. If indicated, participants received injections into both upper and lower limb muscles during the same injection session; a maximum dose of 500 U was given in one session. Muscle localization was undertaken via the use of Teflon-coated injection needles allowing electrical stimulation for muscle localization. Participants in the experimental group then undertook an intensive 8-week rehabilitation program delivered by physiotherapists and occupational therapists. The intensive rehabilitation program consisted of serial casting for contracture reduction, strengthening, and task-specific training according to the principles outlined by Carr and Shepherd [21]. Upper/lower limb casts were applied using procedures previously described by Moseley et al. [22] with the muscle in its maximum obtainable range over the first 2 weeks. Once the final cast was removed, participants received 6 weeks of intensive therapy [23]. Twelve 1-h clinic-based sessions were provided over 6 weeks, with participants undertaking self-directed practice of three 1-h sessions per weekday (each session consisting of 30 min of electrical stimulation and 30 min of task-specific training), i.e., a total of 90 h of self-directed practice. Amount of clinic and home practice was recorded using a paper diary.

Control group 1 received botulinum toxin-A only. Control group 2 received the intensive rehabilitation program only.

### Outcome measures

#### Feasibility

Feasibility of the study involved examining:

- Recruitment
- Adherence, acceptability, and safety of the intervention
- Measurement.

Feasibility of recruitment was determined by calculating the number of enrolled participants as a proportion of the eligible population of adults with spasticity after stroke attending the rehabilitation service and retention of participants. Feasibility of the intensive rehabilitation program was determined by examining adherence to the program. Acceptability was determined from the answer to a question: *which intervention(s) would you prefer given the choice*? Safety was determined by recording injurious events. Feasibility of measurement was determined by being able to measure the clinical outcomes in all participants and recording the time it took.

#### Clinical outcomes

The primary outcome was goal attainment measured using the Goal Attainment Scale [24]. The outcome assessor encouraged the participant to identify their own goals related to the activity and participation in meaningful tasks. Scores (ranging from − 2 to + 2) were given for current and expected levels of performance, and *t*-scores were calculated using the published method of Kiresuk et al. [25].

Secondary outcomes were upper limb activity (for those participants who were identified as having upper limb spasticity) and walking (for those participants who were identified as having lower limb spasticity). Upper limb activity was measured using the Box and Block Test [26] and reported in blocks per second. The Box and Block Test measured unilateral gross manual dexterity and involved the participant moving as many blocks as possible in 60 s. The number of blocks moved was recorded, and a score of 0 was given when the participant was unable to move any of the cubes. Walking was measured using the 6-min walk test [27] and walking speed reported in meters per second. The test was administered as described by Guyatt et al. [27], and participants were able to use walking aids and rest when necessary. Spasticity was measured using the Tardieu Scale [28]. This measure is obtained by moving the joint as fast as possible through its range of movement and rating the quality of the muscle reaction (resistance).

### Sample size rationale

This study was designed as a feasibility trial and is not statistically powered to detect between-group clinically meaningful differences in the primary outcome. One of the study outcomes will be to estimate the variability of the proposed primary and secondary outcomes to inform sample size calculations for future studies. Julious [29] recommends that to establish both feasibility and the precision around the mean and variance (so as to permit future sample size calculations), a minimum of 12 subjects per group should be recruited. Therefore, this feasibility trial aimed to recruit > 12 participants per group, that is, > 36 in total.

### Data analysis

All clinical measures were analyzed using an intention-to-treat analysis approach. Descriptive statistics were calculated for all variables over the three time points (weeks 0, 8, and 12). Within and between-group comparisons of all outcomes are reported as mean difference (95% CI). These exploratory analyses were performed to assess the changes in outcome variables from baseline to 8 and 12 weeks to understand the potential for the interventions to show benefit. Statistical significance was set at 0.05.

## Results

### Characteristics of participants

Thirty-seven adults with spasticity after a neurological condition attending the rehabilitation service participated in the study. The characteristics of participants are presented in Table 1. Twelve participants were allocated to the experimental group, 11 to the control group 1, and 14 to the control group 2. The majority of participants (*n* = 26, 70%) had upper limb spasticity.

**Table 1:** Baseline characteristics of participants

Characteristics	All *n* = 37	Group		
		Experimental group *n* = 12	Control group 1 *n* = 11	Control group 2 *n* = 14
Age (year), mean (SD)	59 (14)	62 (9)	58 (17)	58 (14)
Sex, *n* male (%)	26 (70)	9 (75)	7 (64)	10 (71)
Side of hemiplegia, *n* left (%)	23 (62)	9 (75)	6 (55)	8 (57)
Type of neurological condition, *n* (%)				
Stroke	33 (89)	11 (92)	10 (91)	12 (86)
Multiple sclerosis	1 (3)	1 (8)	0 (0)	0 (0)
Traumatic brain injury	3 (8)	0 (0)	1 (9)	2 (14)
Time post-stroke (*m*th), mean (SD)	50 (54)	36 (49)	80 (69)	38 (37)
Living situation, *n* in nursing home (%)	2 (5)	0 (0)	0 (0)	2 (14)
Spastic limb, *n* upper limb (%)	26 (70)	9 (75)	6 (55)	11 (79)
Goal Attainment Score, *t*-score mean (SD)	23 (0)	23 (0)	23 (0)	23 (0)
Spasticity (Mod Tardieu Scale 0–4), *n* (%)				
(2) Clear catch at a precise angle, interrupting the passive movement, followed by release	28 (76)	7 (58)	10 (91)	11 (79)
(3) Fatigable clonus (10 s when maintaining pressure) occurring at a precise angle	7 (19)	4 (33)	0 (0)	3 (21)
(4) Unfatigable clonus (> 10 s when maintaining pressure) occurring at a precise angle	2 (5)	1 (8)	1 (9)	0 (0)
Pain *(EQ-5D)*, *n* (%)				
None	16 (43)	4 (33)	5 (45)	7 (50)
Moderate	18 (49)	8 (67)	4 (36)	6 (43)
Severe	3 (8)	0 (0)	2 (18)	1 (7)
Mobility (*EQ-5D)*, *n* (%)				
No problems	5 (8)	2 (8)	2 (8)	1 (8)
Some problems	29 (8)	9 (8)	9 (8)	11 (8)
Severe problems	3 (8)	1 (8)	0 (8)	2 (8)
Quality of life (*EQ-5D VAS, 0–100*), mean (SD)	60 (21)	68 (22)	55 (24)	58 (17)

*Exp* experimental group (botulinum toxin-A plus intensive therapy), *Con 1* control group 1 (botulinum toxin-A only), *Con 2* control group 2 (intensive therapy only)

### Feasibility

#### Recruitment

One hundred adults with a neurological condition who were referred to the spasticity clinic of a specialist rehabilitation hospital between September 2010 and September 2011 were screened to participate in the trial with 37 agreeing to participate (eligibility fraction 39%, enrolment fraction 95%). In terms of retention, at week 8, the primary outcome was not collected from three participants—one had withdrawn, one declined, and one was in hospital due to non-study-related medical reasons. At week 12, the primary outcome was not collected from four participants—one had withdrawn and three declined (Fig. 1).

#### Intervention

In terms of botulinum toxin-A, one participant refused botulinum toxin-A injection, citing a preference to receive therapy, and withdrew from the trial. The mean number of total units of Botox™ injected was 232 U (SD 113) while the mean number of units per muscle was 47 U (SD 21) into a mean of 7 (SD 2) muscles (Table 2). In terms of intensive therapy, 24 of 26 participants (92%) allocated to the intensive rehabilitation program received the 2 weeks of serial casting; participants wore their casts for a mean of 13 days (SD 4). Then, participants received 97% of scheduled clinic-based sessions (mean 11 h, SD 2) and recorded a mean of 52 out of 90 planned hours (SD 32, range 5–109) of independent practice. In terms of acceptability, 30 (81%) participants preferred to receive botulinum toxin-A plus intensive therapy, 5 (14%) preferred to receive intensive therapy alone, and 2 (5%) reported no preference; no participant reported a preference for botulinum toxin-A without therapy. In terms of safety, no study-related adverse effects were reported (two participants were hospitalized unrelated to their participation during the period of the trial).

**Table 2:** Summary of botulinum toxin-A injections

Muscle injected	Number of participants	Units per injection
Upper limb		
Flexor carpi radialis	12	25 U, 25 U, 50 U, 50 U, 50 U, 30 U, 50 U, 100 U, 30 U, 50 U, 50 U, 50 U
Flexor carpi ulnaris	12	25 U, 25 U, 50u, 50 U, 50 U, 30 U, 50 U, 100 U, 30 U, 50 U, 50 U, 50 U
Flexor digitorum superficialis	12	25 U, 25 U, 50 U, 40 U, 30 U, 50 U, 30 U, 50 U, 40 U, 25 U, 25 U, 50 U
Flexor digitorum profundis	9	25 U, 25 U, 50 U, 50 U, 30 U, 50 U, 30 U, 60 U, 50 U
Flexor pollicus ongus	3	10 U, 10 U, 10 U,
Flexor pollicus brevis	1	10 U,
Opponens pollicus	1	25 U,
Biceps	8	75 U, 100 U, 30 U, 100 U, 60 U, 100 U, 60 U, 30 U
Brachialis	4	25 U, 100 U, 20 U, 30 U,
Pronator quadratus	3	50 U, 30 U, 50 U
Pronator teres	3	50 U, 30 U, 50 U
Pectoralis major	1	50 U,
Lower limb		
Tibialis posterior	4	70 U, 80 U, 50 U, 50 U,
Extensor hallucis longus	2	30 U, 30 U,
Soleus	4	200 U, 60 U, 50 U, 50 U
Tibialis anterior	3	80 U, 25 U, 50 U
Flexor digiti minimi brevis	2	40 U, 40 U,
Flexor digiti minimi longus	1	40 U,
Gastrocnemius	2	60 U, 100 U,
Adductor magnus	1	20 U
Quadriceps	1	50 U
Iliopsoas	1	50 U

#### Outcome measures

The average time taken to collect the clinical outcomes was 90 min. All outcome measures were able to be collected from 31 (84%) participants across three time points; there were three (8%) participants who consented to only partial assessments at both week 8 and 12.

### Clinical outcomes

Group data for goal attainment, upper limb activity, walking, and spasticity are presented in Table 3. All groups improved during the trial; baseline to 8 and 12 weeks within-group differences in Goal Attainment Scale scores all showed greater than 10% increase, an accepted value for minimum important differences. At week 8, there were also between-group differences: the experimental group had greater Goal Attainment Scale scores than the control group 1 (mean MD 2, 95% CI − 12 to 15) and control group 2 (MD 2, 95% CI − 9 to 13). And by week 12, the experimental group had greater Goal Attainment Scale scores than the control group 1 (MD 4, 95% CI − 9 to 17) and control group 2 (MD 4, 95% CI − 7 to 16). The between-group differences for the Box and Block Test, the 6-min walk test, and Tardieu Scale were near zero with wide confidence intervals. All between-group results are preliminary and should be treated with caution because of the small sample size.

**Table 3:** Mean (SD) of each group, mean (SD) difference within each group, and mean (95% CI) difference between groups

Outcome	Groups									Difference within groups						Difference between groups			
	Week 0			Week 8			Week 12			Week 8 minus week 0			Week 12 minus week 0			Week 8 minus week 0		Week 12 minus week 0	
	Exp	Con 1	Con 2	Exp	Con 1	Con 2	Exp	Con 1	Con 2	Exp	Con 1	Con 2	Exp	Con 1	Con 2	Exp minus Con 1	Exp minus Con 2	Exp minus Con 1	Exp minus Con 2
Goal Attainment Scale *(T-score)*	23 (0) *n* = 12	23 (0) *n* = 11	23 (0) *n* = 14	40 (14) *n* = 12	31 (9) *n* = 9	38 (9) *n* = 12	40 (13) *n* = 12	36 (13) *n* = 8	36 (9) *n* = 12	17 (14)	8 (9)	15 (9)	17 (13)	13 (13)	13 (9)	9 (− 20 to 2)	2 (− 9 to 13)	4 (− 9 to 17)	4 (− 7 to 16)
Box and Block Test*(blocks/s)*	0.14 (0.21) *n* = 9	0.00 (0.00) *n* = 6	0.05 (0.10) *n* = 11	0.13 (0.18) *n* = 9	0.01 (0.01) *n* = 5	0.07 (0.16) *n* = 11	0.12 (0.19) *n* = 9	0.02 (0.04) *n* = 5	0.07 (0.15) *n* = 11	− 0.01 (0.05)	0.01 (0.01)	0.02 (0.07)	− 0.02 (0.06)	0.02 (0.04)	0.02 (0.07)	− 0.02 (− 0.06 to 0.03)	− 0.03 (− 0.09 to 0.03)	− 0.04 (− 0.10 to 0.03)	− 0.04 (− 0.10 to 0.02)
6-min Walk Test*(m/s)*	0.18 (0.16) *n* = 3	0.54 (0.05) *n* = 5	0.46 (0.58) *n* = 3	0.28 (0.24) *n* = 3	0.61 (0.17) *n* = 4	0.46 (0.53) *n* = 3	0.27 (0.23) *n* = 3	0.62 (0.29) *n* = 4	0.35 (0.60) *n* = 3	0.10 (0.09)	0.07 (0.19)	0.01 (0.09)	0.09 (0.09)	0.08 (0.30)	− 0.11 (0.13)	0.02 (− 0.29 to 0.33)	0.09 (− 0.12 to 0.29)	0.01 (− 0.46 to 0.48)	0.20 (− 0.05 to 0.44)
Tardieu Scale*(0–4)*	2.5 (0.7) *n* = 12	2.2 (0.4) *n* = 11	2.2 (0.6) *n* = 14	2.5 (0.7) *n* = 11	2.3 (0.5) *n* = 9	2.0 (0.5) *n* = 13	2.3 (0.7) *n* = 12	2.2 (0.4) *n* = 9	2.2 (0.8) *n* = 12	− 0.1 (0.3)	− 0.2 (0.8)	0.1 (0.3)	− 0.2 (0.4)	0.0 (1.1)	0.0 (0.0)	0.1 (− 0.4 to 0.7)	− 0.2(− 0.4 to 0.1)	− 0.2 (− 0.9 to 0.6)	−20090.2 (− 0.4 to 0.1)

*Exp* experimental group (botulinum toxin-A plus intensive therapy), *Con 1* control group 1 (botulinum toxin-A only), *Con 2* control group 2 (intensive therapy only)

## Discussion

The addition of 8 weeks of intensive therapy based on best-available evidence and delivered as structured clinic and home-based outpatient therapy appears feasible for adults with neurological spasticity. Participants attended the majority of sessions, complied with the home-practice program and reported no adverse effects from the intensive therapy program or botulinum toxin injections. Only one participant withdrew from the intensive physiotherapy program for non-study-related reasons. This study found that it is feasible to provide intensive therapy, and while there were promising improvements noted within groups suggesting potential for the intervention to enable change, there were no benefits of one intervention relative to another in terms of goal achievement, upper limb activity, or walking.

In current clinical practice, adults with spasticity who attend an Australian spasticity clinic are more likely to receive botulinum toxin-A alone rather than botulinum toxin-A followed by intensive rehabilitation [30]. Within-group changes suggest potential benefits to activity and goal attainment in favor of intensive rehabilitation; however, between-group differences were small and not statistically significant. This was a feasibility study with only 37 participants; therefore, effects should be tested in a study with a sufficient sample size. Our adherence rate was also moderate at best, and future studies that test exercise programs carried out largely at home should include aspects in their design that focus on promoting adherence (e.g., learner contracts, motivational interviewing [31], structure and support [32], or use of a standardized home program such as the Graded Repetitive Arm Supplementary Program (GRASP) [33]). This study found no difference either within or between groups on the measure of spasticity (Tardieu scores) at 12 weeks. Consistent with previous research [34], our findings suggest that the effects of botulinum toxin-A had worn off by this time point. We recommend that future trials determining the additive effects of therapies continue to re-measure variables (i.e., follow-up) at 12 weeks post-injection when the effects of botulinum toxin-A have dissipated.

In adults with spasticity, there are large variations in spasticity severity, limbs affected, and how much the spasticity interferes with the person’s ability to move. It is, therefore, important to define the population to which this study’s findings apply. This study included all people with spasticity that interfered with meaningful movement (upper or lower limb), although in reality, the majority of referred participants had upper limb spasticity (70%). Between-group differences on the secondary outcomes of upper limb activity and walking demonstrated wide confidence intervals and do not provide sufficient support for implementing this rehabilitation program in such a mixed group without further research. With fewer numbers of patients presenting with lower limb spasticity, outcomes on the 6-min walk test should be interpreted with caution, and future trials may need to focus on either the upper or the lower limb in order to ensure sufficient representation in each group.

There are some limitations of this study. The study sample was heterogeneous in nature, and while the primary outcome of goal attainment was able to demonstrate benefit of prescribing therapy to patients after botulinum toxin-A, it was not necessarily able to demonstrate a benefit of prescribing botulinum toxin-A to patients who are receiving intensive therapy interventions. The planned sample size of the current feasibility study was small, and the population varied across the sample with respect to motor ability as is typical of a pilot trial. To reduce heterogeneity, future studies should use baseline ability (walking or upper limb activity) to stratify groups prior to randomization. Selecting only participants with either upper or lower limb spasticity would also reduce the notable heterogeneity. We additionally acknowledge the moderate adherence to the home practice component of our therapy program. Strategies to increase adherence to practice should be embedded in future trials.

## Conclusion

This study has successfully demonstrated the feasibility of a trial that involves prescribing an intensive dose of therapy intervention to outpatients with a neurological injury or illness. While this study was underpowered, power analysis for a future study using Goal Attainment Scale data of the current study (SD 13.75) suggests that a sample size of 136 participants (68 in each group) would be necessary to detect a between-group difference of 7 points on Goal Attainment Scale *t*-score with an alpha of 0.05 and power of 0.80. Given the number of goals set, a 7-point difference represents approximately 10% improvement; clinically, this would equate to achieving at least one goal or partially achieving at least two goals.

In summary, providing an intensive home and clinic-based therapy program to patients after they have received botulinum toxin-A injections for spasticity management appears feasible. In addition, the experimental group demonstrated greater achievement of goals than either control group, particularly at the end of 3 months when the effect of botulinum toxin-A had begun to wane. A larger trial to understand the benefits of delivering the intensive therapy after botulinum toxin-A with respect to upper limb activity or walking is therefore warranted.

## Abbreviations

6-min walk test: 6 min walk test
Con: Control
EQ-5D: European quality of life-5 dimension
Exp: Experimental
GRASP: Graded repetitive arm supplementary program
MD: Mean difference
mod Tardieu scale: Modified tardieu scale
n: Number
SD: Standard deviation
VAS: Visual analogue scale
